# Early electrophysiological study variants and their relationship with clinical presentation and outcomes of patients with Guillain-Barré syndrome

**DOI:** 10.1038/s41598-023-41072-x

**Published:** 2023-08-26

**Authors:** Eman M. Khedr, Mohamed M. Shehab, Mohamed Z. Mohamed, Khaled O. Mohamed

**Affiliations:** Department of Neurology and Psychiatry, Faculty of Medicine, Assiut University Hospital, Assiut, Egypt

**Keywords:** Biomarkers, Diseases, Neurology

## Abstract

This study compared the clinical outcomes of the two main neurophysiological types of Guillain-Barré Syndrome (GBS). Sixty-two GBS patients were examined clinically at onset using Medical Research Council (MRC), Hughes disability scales (HDS), and nerve conduction studies were evaluated in four limbs. The Modified Erasmus GBS outcome score (MEGOS) was assessed 2 weeks after onset. Outcomes were measured after 3 months using MRC and HDS scores. According to electrophysiological data two main groups identified acute inflammatory demyelinating polyneuropathy (AIDP = 31 cases) or acute axonal GBS including inexcitable forms (26 cases). The number of days between onset of weakness and admission was significantly shorter, and gastrointestinal symptoms were significantly higher among the axonal type than AIDP. MRC sum scores at onset and at nadir were significantly worse in the axonal type than in AIDP. Neck muscle weakness, impaired cough reflex, the need for mechanical ventilation, hypoalbuminemia, and hypernatremia were more common in the axonal type. At outcome, 74% of the AIDP were healthy/minor symptoms versus 38.46% of the axonal type. There was a high prevalence of the axonal variant (41.9%) compared with European and North American populations. The axonal type had a significantly worse outcome than AIDP type.

## Introduction

Guillain-Barré Syndrome (GBS) is a heterogeneous, immune mediated polyradiculoneuropathy. The main forms are acute inflammatory demyelinating polyradiculoneuropathy (AIDP), acute motor axonal neuropathy (AMAN), and acute sensorimotor axonal neuropathy (ASMAN). Each form has unique clinical, pathological and pathophysiological features^1^.

The clinical spectrum ranges from mild symptoms to severe, rapidly progressing weakness with life threatening sequels (as respiratory paralysis and autonomic dysfunction)^2,3^. It has been demonstrated that an early diagnosis reduces the morbidity and improves the prognosis of the disease^4^.

GBS diagnosis is usually clinical and requires the presence of progressive motor weakness of more than one limb and areflexia^5^. Diagnosis is supported by electrodiagnosis (EDx), including nerve conduction studies (NCS) and electromyography (EMG). EDx findings consistent with polyneuropathy are obligatory to fulfill the criteria for level 1 diagnostic certainty according to the clinical case definition of the Brighton Collaboration GBS Working Group^6^.

Electrophysiological subtypes according to Rajabally’s criteria are acute inflammatory demyelinating polyradiculoneuropathy (AIDP), axonal GBS including inexcitable forms, and equivocal^7^.

Functional outcome and mortality are affected by the variant of GBS. Several studies have reported increased mortality and morbidity in the axonal variant^8,9^.

Functional outcome after GBS can be assessed using a number of different clinical scales such as the Medical Research Council (MRC) sum score^10–12^, Hughes Disability Scale (HDS)^13^ and Modified Erasmus GBS outcome score (MEGOS)^7,14^.

Up to our Knowledge there was no published data from Egypt about associations between electrophysiology variants and clinical presentation/outcomes of GBS. This study was conducted to examine the clinical presentation and functional outcomes in early neurophysiological variants of GBS.

## Results

This study included 62 patients who were classified according to Rajabally’s et al.^7^ criteria into: acute demyelinating polyneuropathy (31 patients AIDP variant), acute axonal GBS including inexcitable forms (AMAN + ASMAN + Inexcitable variants) in 26 cases, and equivocal (5 patients). Because the number of latter groups was small, we only compared results in the two main groups.

### Demographic and clinical data

Demographic and clinical data in the main electrophysiological variants (AIDP and Primary Axonal type) at admission are detailed in Table 1. There were no significant differences between the two groups in demographic data. The number of cases with antecedent events was significantly higher among axonal variant than AIDP. Gastrointestinal symptom (antecedent event) was significantly more common in the axonal type than AIDP whereas upper respiratory tract infection was more common in AIDP. Concerning the number of days between onset of symptoms and electrophysiological study; most of the patients located in early (> 10 days) and late stage (5–10 days) with no significant differences between AIDP and axonal variants. The number of days between onset of weakness and admission was significantly shorter in the axonal type than AIDP. MRC sum scores at onset and at nadir were significantly worse, and neck muscle weakness was more common, in the axonal type. In contrast, sensory symptoms were more common in AIDP. EGRIS showed that patients in the axonal group had a significantly higher intermediate and higher risk for mechanical ventilation than those with AIDP.

**Table 1:** Demographic and clinical data in relation to neurophysiological variants at admission.

Clinical findings at onset	AIDP	Primary axonal including inexcitable forms	*p* value*
	(N = 31)	(N = 26)	
Demographic data			
Age: mean SD (range of age years)	36.81 ± 18.31	34.24 ± 16.13	0.58
Sex: Male/Female Number (%)	18/13(58.1/41.9%)	14/12(53.8/46.2%)	0.74
Residency: Urban/Rural Number (%)	14/17(45.2/54.8%)	8/18(30.8/69.2%)	0.27
Duration of each event			
Days between antecedent event and onset of GBS: Mean ± SD	8.26 ± 7.57	7.58 ± 7	0.72
Duration of antecedent event: Mean ± SD	1.84 ± 1.61	1.85 ± 1.51	0.98
Days between onset of weakness and admission: Mean ± SD	8.1 ± 5.04	5.35 ± 3.63	0.02
Number of cases with antecedent events = 39 (68.4%)	17(54.8%)	22(84.6%)	0.016
Upper RT infection ± fever: Number (%)	14(82.35%)	12(54.54%)	0.94
GIT infection: Diarrhea ± vomiting: Number (%)	3(17.6%)	10(45.45%)	0.01
Days between onset and admission			
> 7 days	14(45.2%)	7(26.9%)	0.15
4–7 days	7(22.6%)	4(15.3%)	
≤ 3 days	10(32.3%)	15(57.6%)	
Days between onset and electrophysiology study			
> 10–15 days	15(48.4%)	6(23.1%)	0.14
5–10 days	15(48.4%)	19(73.1%)	
≤ 4 days	1(3.2%)	1(3.8%)	
Cranial nerves affection			
Presence	18(58.1%)	19(73.07%)	0.23
Absence	13(41.9%)	7(26.9%)	
Papilledema and blurred vision			
Present	5(16.1%)	8(30.7%)	0.19
Absent	26(83.9%)	18(69.2%)	
Neck muscle			
Affected	9(29%)	17(65.38%)	< 0.006
Not affected	22(71%)	9(34.6%)	
Sensory affection			
Hypoesthesia	29(93.5%)	13(50%)	< 0.0001
Intact sensation	2(6.5%)	13(50%)	
Cough reflex			
Impaired cough reflex	12(38.7%)	18(69.2%)	0.022
Preserved	19(61.3%)	8(30.7%)	
MRC sum score			
At onset Mean ± SD	27.16 ± 13.96	15.15 ± 11.45	0.001
At nadir Mean ± SD	31.16 ± 13.93	18.23 ± 13.43	0.001
EGRIS at onset			
Low risk	11(35.5%)	0	0.001
Intermediate risk	7(22.6%)	7(26.9%)	
High risk	13(41.9%)	19(73.07%)	
Mechanical ventilation			
Mechanical ventilated	0	4(15.3%)	0.024
Not ventilated	31(100%)	22(84.6%)	
Autonomic affection (dysautonomia)			
Present	7(22.6%)	10(38.4%)	0.192
Absence	24(77.4%)	16(61.5%)	
Treatment			
Plasma exchange	24(77.4%)	22(84.6%)	0.49
IVIG	7(22.6%)	4(15.3%)	

*RT respiratory tract, *GIT* gastrointestinal; *EGRIS* erasmus guillain barre respiratory insufficiency score risk.

GBS subtyping according to Rajabally’s criteria in relation to the time passed from onset of illness to electrophysiology study is illustrated (Table 2) which shows that; only two patients (3.2%) located in very early stage, most of the primary axonal type, Equivocal, and nearly half of AIDP had the electrophysiological study during early stage 5–10 days from the onset of illness (59.7%), while 37.1% underwent electrophysiological study at late stage > 10 days.

**Table 2:** GBS subtyping following Rajabally’s criteria in relation to the time passed from onset of illness to electrophysiology study.

Staging for electrophysiological study	AIDP Number of patients (31 cases)	Primary axonal type (26 cases)			Equivocal number of patients	Total number of patients (%)
		AMAN number of patients	AMSAN number of patients	Inexcitable number of patients		
Very early stage 1–4 days	1	1	0	0	0	2 (3.2%)
Early stage 5–10 days	15	9	5	5	3	37 (59.7%)
Late stage > 10 days	15	2	4	0	2	23 (37.1%)

*AIDP* acute inflammatory demyelinating polyradiculoneuropathy, *AMAN* acute motor axonal neuropathy, *AMSAN* acute motor and sensory axonal neuropathy.

### Laboratory findings

Laboratory findings in relation to electrophysiological variants are shown in Table 3. Respiratory acidosis at onset was more common in the axonal type as were hypoalbuminemia and hyponatremia. Other laboratory data were not significantly different.

**Table 3:** Laboratory findings of in relation to neurophysiological variants at admission.

Laboratory findings (Normal range)	AIDP (N = 31)	Primary axonal including inexcitable forms (N = 26)	*p* value*
Total leukocyte count TLC (4000–10,000/L)			
Within normal	11 (35%)	11 (42.3%)	0.59
Leukocytosis	20 (64.5%)	15 (57.6%)	
Serum protein level (64–83 g/l)			
Within normal	22 (71%)	17 (65.3%)	0.65
Hypoproteinemia	9 (29%)	9 (34.6%)	
Serum albumin level (34–50 g/l)			
Within normal	23 (74.2%)	17 (65.3%)	0.46
Hypoalbuminemia	8 (25.8%)	9 (34.6%)	
C-reactive protein (CRP)			
Negative	19 (61.3%)	15 (57.6%)	0.78
Elevated	12 (38.7%)	11 (42.3%)	
Serum sodium NA level (136–145 mmol/L)			
Within normal	23 (74.2%)	14 (53.85%)	0.03
Hyponatremia	8 (25.8%)	12 (46.15%)	
Cytoalbumious dissociation in CSF (42 cases only)			
Negative	8 (44.4%)	7 (33.3%)	0.47
Positive	10 (55.6%)	14 (66.6%)	
Mean ± SD (Range)	157.32 ± 76.04 (58 to 264)	139.29 ± 40.48 (88 to 210)	0.51#
Arterial blood gases			
Respiratory acidosis	1 (3.22%)	5 (19.2%)	0.05
Normal ABG	30 (96.7%)	21 (80.67%)	

^#^ Independent T-test.

### Clinical outcome scores

Clinical outcomes in the two electrophysiological groups are shown in Tables 4 and 5. Patients in the axonal group had significantly worse outcomes in the MRC sum score, HDS, and MEGOS scales. 84% of AIDP patients achieved a normal (full strength) MRC SS equal to grade 0 compared with only 50% in the axonal type. Six cases in the axonal group (23%) had an MRC grade of 3 to 4 (score < 20) versus none in the AIDP group. 74% of patients in the AIDP group became healthy or had only minor symptoms versus 38.5% in the axonal group. There was only one death (3.2%) in the AIDP group versus 8 (30.7%) who were bedridden or dead (5 cases) in the axonal group.

**Table 4:** Clinical rating Scale after 3 months of treatment in relation to Electrophysiological variants at Baseline assessment.

Outcome scales after 3 months of treatment	AIDP (N = 31)	Primary axonal including inexcitable forms (N = 26)	*p* value by one way ANOVA
MRC sum score	56.5 ± 6.5	44.48 ± 17.7	df = 54, F = 21.82, *p* < 0.0001
HDS	1.29 ± 0.5	2.23 ± 1.86	df = 55, F = 11.76, *p* = 0.001
MEGOS	1.94 ± 1.2	2.65 ± 2.15	df = 55, F = 4.58, *p* = .0.037

*MRC* medical research council, *HDS* hughes disability scale, *MEGOS* modified erasmus GBS outcome score.

**Table 5:** Clinical rating Scale grading after 3 months of treatment in relation to electrophysiological variants at baseline assessment.

Different grading of clinical score at 3 months follow up	AIDP (N = 31)	Primary Axonal including Inexcitable forms (N = 26)*	*p* value
MRC sum score (MRC SS) and grade			
MRC SS 51–60 = Grade 0	26 (83.9%)	12 (46.15%)	< 0.0001**
MRC SS 41–50 = Grade 1	4 (12.9%)	4 (15.38%)	
MRC SS 31–40 = Grade 2	1 (3.2%)	4 (15.38%)	
MRC SS 21–30 = Grade 3	–	3 (11.53%)	
MRC SS < 20 = Grade 4	–	3 (11.53%)	
HDS grade			
0–1 Healthy/minor symptoms	23 (74.2%)	10 (38.46%)	0.001
2–3 Walk ± support	7 (22.6%)	8 (30.7%)	
4–6 Bed ridden ± assisted ventilation or dead	1 (3.2%)	8 (30.7%)	

*MRC* medical research council score, *HDS* hughes disability scale.

*1 dead from axonal type.

**p value by Pearson chi-square.

## Discussion

### Electrophysiological classification

The goal of this study was to compare the outcomes of the two main electrophysiological variants of GBS at three months’ follow-up.

Several electrophysiological criteria have been proposed for classification of patients with GBS^15–18^. An electrophysiological study using Ho’s criteria^19^ or Hadden’s criteria^15^ for GBS was inadequate for accurate diagnosis of GBS subtype due to the lack of specificity of cut-off values for motor DL and MCV^20^. Furthermore, these criteria do not allow appropriate recognition of the various forms of axonal GBS^21^. Nedkova et al.^22^ reported that even using optimized electrophysiological criteria, accurate subtyping is only possible in a minority of very early GBS cases; however in the present study electrophysiology was performed in most of cases during 5–10 and > 10 days from the onset of symptoms (60 cases), and only two patients had electrophysiology study very early ≤ 4 days. Rajabally’s electrophysiological criteria make it possible to diagnose GBS subtype earlier and accurately with a single nerve conduction study. With Rajabally’s criteria, the proportion of equivocal cases is reduced, resulting in an improved sensitivity for a non-equivocal diagnosis of GBS, that is, of AIDP or axonal GBS. This is helpful as it offers a greater probability of reaching a firm classification within the first 7 days from the onset with a single electrophysiological study^7^.

Regional differences exist in the distribution of various GBS variants^23^. Up to 90% of instances in Europe and North America are of the demyelinating variety, and less than 10% are of the axonal variety^1,24^, whereas in Asia the incidence of the axonal subtype ranges from 30 to 65%^9,25–27^. In the present study 42% of patients were of the axonal subtype whereas 50% had AIDP, which is similar to previous in large studies in China, Japan and South America^25,26^. Our findings are a representative of Upper Egypt as Assiut City is the biggest governorate in Upper Egypt and patients come from all areas of Upper Egypt. In order for our findings to be reflective of Egypt as a whole, we will need to replicate the existing data through the future conduction of a multicentric national research with a bigger sample size.

The geographical variations of electrophysiological variants in GBS have several possible explanations. First it could be explained by variations in the antecedent infection (precipitating antigen) or variations in the immune responses to infectious pathogens, which produce antibodies against various peripheral nerve epitopes^1^. Second, the variability in the definition of AIDP and axonal type may influence diagnosis. Third, the timing of the NCS could impact the results.

### Relationship between electrophysiological categories and clinical presentation at the onset of illness

Our results provide new insights into the relationships between neurophysiological categories and clinical features at the time of electrophysiological testing.

In the present study there were no significant differences between AIDP and axonal subtypes in age, sex and residence. The number of days between onset of weakness and admission was significantly shorter in the axonal type than AIDP and MRC sum scores at onset and at nadir were significantly worse, in the axonal type than AIDP. This shorter duration with rapidly evolving weakness in axonal variant can be supported by the results of autopsy studies in early GBS and also in pathologically well-illustrated models of experimental autoimmune neuritis (EAN); they have revealed that the initial histological background is inflammatory edema predominating in proximal nerve trunks, and particularly in spinal nerves; intriguingly this notion is applicable to AMAN and this was confirmed by delayed late response (F wave and H reflex) or absent in inexcitable variant in early stage (5–10 days) in the present study. Demyelination and primary or secondary axonal degeneration usually appears into the scene later on^28^. Bearing these notions in mind, it will come as no surprise that the most frequent early electrophysiological features are late response alteration and CMAP attenuation.

According to electrophysiological data, acute axonal GBS included inexcitable forms was recorded in 26 cases. In the chronology of nerve inexcitability: Wallerian degeneration motor-evoked responses are reduced by 50% at 3–5 days after nerve injury, the response being absent by day 9^29^. In the present study, the electrophysiology study was done in most of primary axonal type (9 cases AMAN, 5 cases ASMAN, and 5 cases inexcitable forms) during early stage (5–10 days from the onset of illness) and 6 cases (4 cases AMAN, and 2 cases AMSAN) during late stage (≥ 10 days). In very early GBS (≤ 4 days) an alternative mechanism of nerve inexcitability might be endoneurial ischemia caused by inflammatory edema in nerve trunks possessing epi-perineurium^29^ however we had no patients with inexcitable nerve that was recorded in the very early stage (≤ 4 days).

### Antecedent events were more common among the axonal type than in AIDP

The percentage of patients with antecedent infection (68.4%) was slightly lower than that reported by Doets et al.^23^. They reported antecedent events before neurological onset in 649 (76%) in GBS mainly upper respiratory tract infections (35%) and gastroenteritis (27%)^23^. Fever and upper respiratory tract infection were more common among patients with AIDP while GIT symptoms (diarrhea and vomiting) were more common among patients with the axonal type. Preceding repeated vomiting and diarrhea in patients with the axonal type could be explaining the associated higher rates of hypoalbuminemia and hyponatremia. Moreover, it may be related to Campylobacter jejuni (C. jejuni) infection, which frequently precedes GBS and is associated with axonal degeneration^30^; as C. jejuni infection usually have diarrhea, fever and nausea and vomiting may accompany the diarrhea. However, confirmation of this explanation requires testing for C. jejuni and ganglioside antibodies. Unfortunately the test is unavailable in our region. The pathogenetic processes of axonal GBS associated with bacterial or viral illnesses other than C jejuni remain unclear^31^. Preceding diarrhea was associated with worse outcome in one study^32^ and high rate of hypoalbuminemia in the axonal group also was associated with worse clinical presentation and prognosis. This is in agreement with the study of Fokkink et al.^33^ who found a significant association between hypoalbuminemia and severity of weakness at nadir and the need for mechanical ventilation. Approximately 65% of those with hypoalbuminemia could walk unaided after 6 months compared with 90% of those with normal serum albumin^34^. Hypoalbuminemia is associated with a poor prognosis in many diseases and after surgery^35^. Serum albumin may be a leading candidate biomarker for outcome in GBS^34^.

Albumin loss by extravasation into the inflamed nerves is a possible cause of hypoalbuminemia, because in severe cases (axonal type) cerebrospinal fluid protein rises to more than one gram per liter with similar extravasation throughout the peripheral nervous system. Hemodilution may also contribute since it occurs with mechanical ventilation, fluid replacement and recumbence, which are common in GBS^34^. Consistent with this, we observed a higher rate of mechanical ventilation in patients with the axonal type while no patients were ventilated in the AIDP group.

In the present study MRC sum scores at onset and at nadir were significantly worse and neck muscle weakness more common in the axonal type than AIDP. In contrast, sensory dysfunction was significantly higher in the AIDP group than in the axonal group. This contrasts with results in two large electrophysiological studies that found no correlation between the severity of motor weakness and electrophysiological subtype^15,36^.

The present results showed that impaired cough reflex was significantly more common in the axonal type than AIDP. According to the EGRIS score, patients in the axonal group had a significantly higher intermediate/high risk of respiratory insufficiency needing mechanical ventilation than in AIDP. The increased risk of respiratory impairment could the higher percentage of respiratory acidosis in patients with the axonal type.

In contrast, Ho et al.^37^ found no difference in the rates of respiratory insufficiency between patients with axonal GBS (acute motor axonal neuropathy, AMAN) and AIDP.

### Outcome

The worst outcomes occurred in patients with the axonal variant. Similarly, a study by Akbayram found increased morbidity and mortality in the axonal variant^8^. Other studies found that dCMAP amplitude < 20% of the lower limit of normal was the most powerful predictor of poor outcome^38–40^. Such results were expected because Wallerian degeneration has long been considered to have a worse outcome than demyelination. However our findings are inconsistent with other reports in which a considerable number of patients with the axonal variant of GBS recovered quickly and even more rapidly than patients with demyelinating variant^37,41^. In other reports, motor disability at outcome was similar in both electrophysiological groups^15,36^.

These contradictory findings can be explained by the fact that the axonal variant of GBS may either improve very slowly and incompletely or may recover rapidly and fully, even more rapidly than demyelinating variant^42^. The possible mechanism of slow and incomplete recovery in most cases of axonal GBS is secondary axonal degeneration. In these patients, full motor recovery requires axonal regeneration which is a slow and incomplete process^39^. On the other hand, rapid recovery in some cases of axonal GBS could be explained by rapid recovery of early reversible conduction block^21,41^. In these cases, functional conduction block could be caused by deposition of immunoglobulin, complement, and macrophages at the nodes of Ranvier leading to its lengthening and distortion of paranodal myelin^43,44^. Another possible mechanism involves antigangliosides GMI antibodies, which are frequently detected in the sera of AMAN patients^19,45^, that impair sodium and potassium channel function in voltage-clamped single fibers^46^. Finally, A Chinese study suggested that one possible explanation of rapid recovery was that degeneration was limited to intramuscular motor terminal nerves and loss of innervation at the neuromuscular junction^37^.

Most of the patients 32 (52%) in this study were treated with plasmapheresis, which is comparable to other local studies done by Yakoob et al.^47^. Although one of the RCTs done by Asghar et al. on plasmapheresis versus IVIg showed significant improvement in mean disability score at four weeks in patients treated with IVIg (p0.05). Studies showed that intravenous immunoglobulin (IVIg) and plasma exchange are both effective treatments in GBS, IVIg is the preferred treatment only for practical reasons^48,49^. However, supportive care is still the most important component of management^49^.

## Limitation of the study

The small sample size of this study is one of the limitations. Because C jejuni infection frequently precedes GBS particularly in axonal variant, absence of testing for C. jejuni and ganglioside antibodies is also one of the limitations of this study. Further multicenter study to be a representative for Egypt with a higher sample size, repeated electrophysiology in verly stage and measuring C. jejuni and ganglioside antibodies is recommended to confirm the results of the present study.

## Conclusions

We conclude that there is a high prevalence of the axonal variant (41.9%) in the Egyptian population. Patients with the axonal type had a significantly worse clinical presentation and outcome as compared with patients with AIDP in which 85% had good outcome. Most patients were treated with PE, although there was no significant difference in the mean improvement of the axonal or AIDP group.

## Methods

The study included 62 patients with Guillain-Barré syndrome diagnosed according to criteria of the National Institute of Neurological Disorders and Stroke (NINDS) (revised form 1990)^50^ and the Brighton Collaboration in 2014^51^. The study was performed during the period from first of September 2020 to end of October 2021 at Assiut University Hospital, Egypt. They were consecutively recruited from inpatients and ICU immediately after admission within the first 2 weeks of onset of illness.

*Inclusion criteria* Acute onset of progressive symmetrical bilateral weakness of four limbs; absent or decreased tendon reflexes with mild sensory symptoms, cranial nerve affection, dysautonomia; features that support a diagnosis of GBS with progression of no more than four weeks.

*Exclusion criteria* Atypical GBS, age younger than 10 years or older than 75 years; any systemic disease affecting peripheral nerves (Diabetes Mellitus, collagen disease, endocrinal or malignancy); serious pre-existing disease; refusal to undergo electrophysiological testing.

On admission all patients received the following assessments:

*Clinical examination* Age, sex, time of symptom onset and presence of other co-morbidities or preceding antecedent events all. Vital signs including blood pressure, heart and respiratory rates, as well as arterial blood gases (ABG) were also recorded.

### Medial research council (MRC) sum score

This clinical grading scale evaluates strength in three muscle groups of all four limbs. A score between 0 and 5 is assigned to each of them, which gives a maximum total score of 60^10–12^. Scores were assessed at onset and at nadir**.**

### Hughes disability scale (HDS)

This has six levels: 0 points (healthy), 1 point (minor symptoms and capable of running), 2 points (able to walk 10 m without assistance but unable to run), 3 points (able to walk 10 m across an open space with help), 4 points (bedridden or wheelchair-bound), 5points (requiring assisted ventilation for at least part of the day), and 6 points (dead)^13^.

### Erasmus Guillain barre respiratory insufficiency score (EGRIS) risk

It estimates the risk of respiratory failure. At admission and according to EGRIS, three clinical factors are explored: the time from onset of weakness to admission, presence of facial and/or bulbar weakness, and severity of muscle weakness according to MRC sum score^14^. In the EGRIS scale, the MRC sum score was categorized into five categories that ranged from 0 to 5 (MRC Sum Score 60–51 = Grade 0; 50–41 = Grade 1; 40–31 = Grade 2; 30–21 = Grade 3; < 20 = Grade 4) with 0 for higher sum score totals and 5 for the lowest (weakest)^52^.

*Neurophysiology* Electrophysiological measures were obtained within 48–72 h of admission. A Nihon Kohden 9400 machine (Japan) was used to measure motor distal latency (MDL -m/s), distal compound muscle action potential (dCMAP) amplitude ((from peak to peak in mV), motor conduction velocity (dMCV (m/s), sensory distal latency SDL (ms), sensory amplitude (uV), sensory conduction velocity (SCV-m/S), F-wave latency (ms) of median, ulnar, posterior tibial (PTN) and common peroneal nerve (CPN)^53^. F-response (ms) was recorded in five motor nerves (median, ulnar, common peroneal and tibial). All nerves were measured bilaterally and compared with data from 40 normal age and sex matched volunteers. Distal stimulation was applied at the wrist and ankle, and proximal stimulation at the elbow and knee. Conduction velocities were calculated in the elbow-to-wrist and knee-to-ankle segments. Repeated supramaximal stimulations were applied to elicit F-responses. Electrophysiological testing of sensory nerves and needle electromyography were performed in all patients. Sensory nerve potentials were recorded at the median, ulnar, sural, and right and left peroneal nerves. Distal latencies, conduction velocities, and sensory nerve action potentials were analyzed. Concerning number of days between onset of symptoms and electrophysiological study, patients was classified according to Nedkova et al.^22^ into very early (≥ 4 days) in 2 patients, early (< 10 days) in 21 patients and late (> 10 days) in 34 patients. According to Rajabally criteria^7^ Acute inflammatory demyelinating polyradiculoneuropathy (AIDP) was diagnosed if one the following is present in 2 nerves (MCV < 70% lower limb nerve (LLN), DML > 150% upper limb nerve (ULN), F-response latency > 120% ULN, or > 150%, ULN (if dCMAP < 50% of LLN); absence F-wave with dCMAP 20% LLN. Axonal GBS including inexcitable forms was diagnosed if one the following is present in 2 nerves (dCMAP < 80% LLN; absence F-wave with distal CMAP ≥ 20% LLN; proximal compound action potential (pCMAP)/dCMAP amplitude ratio < 0.7; absence F-wave in 1 nerve with dCMAP ≥ 20% LLN or pCMAP/dCMAP amplitude ratio < 0.7 in 1 nerve; and in addition, dCMAP < 80% LLN in 1 other nerve). Equivocal: Abnormal findings not fitting criteria for any other subtype. Because the number of patients with criteria of last group was small (5 cases) we excluded this group.

*Modified Erasmus GBS outcome score (MEGOS) two weeks after admission* This predicts walking inability risk in patients with GBS. It ranges from 0 to 9 with 4 categories for the MRC sum score, 3 categories for age, and 2 categories for preceding diarrhea^7,14^.

*Functional assessment after 3 months of treatment* Medial research council (MRC) sum score, Hughes disability scale (HDS), and Modified Erasmus GBS outcome score (MEGOS).

### Treatment

All patients received treatment immediately after diagnosis either by Plasmapheresis (plasma exchange with 5 sessions given one every other day) or intravenous immunoglobulin (IVIG) 0.4 g/kg/day for 5 consequative days (according to availability of treatment).

### Statistical analysis

All data were analyzed with the aid of the SPSS version 16. Continuous data were expressed as mean ± SD and categorical data were expressed as number (percent). Shapiro–Wilk Normality Test was used to assess the normal distribution of data. As there was no differnce between neurophysiological findings between right and leftt limbs, we combined the data in the upper and lower limbs. Student’s t-test and one-way ANOVA were used for numerical data and Chi-square for non-numerical data to assess the statistical significance of difference between groups. Pearson correlation coefficient was used to assess correlations.

### Ethical approval

Informed written consent was obtained from all the patients or their relatives after approval of Ethical Committee of Faculty of Medicine, Assiut University. The Ethical approval number: 17101401 with register on Clinicaltrials.gov ID: NCT04927598 at 8 April 2021. The confidentiality of patients’ information was maintained during all steps of the study. The research design adheres to the ethical principles outlined in the Helsinki Declaration of 1975.

## Data Availability

Data can be made available to qualified investigators upon reasonable request to the corresponding author.
